# Non-invasive MRI Assessments of Tissue Microstructures and Macromolecules in the Eye upon Biomechanical or Biochemical Modulation

**DOI:** 10.1038/srep32080

**Published:** 2016-08-26

**Authors:** Leon C. Ho, Ian A. Sigal, Ning-Jiun Jan, Xiaoling Yang, Yolandi van der Merwe, Yu Yu, Ying Chau, Christopher K. Leung, Ian P. Conner, Tao Jin, Ed X. Wu, Seong-Gi Kim, Gadi Wollstein, Joel S. Schuman, Kevin C. Chan

**Affiliations:** 1 NeuroImaging Laboratory , University of Pittsburgh, Pittsburgh, PA, USA; 2 UPMC Eye Center, Ophthalmology and Visual Science Research Center, Department of Ophthalmology, School of Medicine, University of Pittsburgh, Pittsburgh, PA, USA; 3 Department of Electrical and Electronic Engineering, The University of Hong Kong, Hong Kong, China; 4 Department of Bioengineering, Swanson School of Engineering, University of Pittsburgh, Pittsburgh, PA, USA; 5 McGowan Institute for Regenerative Medicine, University of Pittsburgh, Pittsburgh, PA, USA; 6 Louis J. Fox Center for Vision Restoration, University of Pittsburgh, Pittsburgh, PA, USA; 7 Department of Chemical and Biomolecular Engineering, Hong Kong University of Science and Technology, Hong Kong, China; 8 Division of Biomedical Engineering, Hong Kong University of Science and Technology, Hong Kong, China; 9 University Eye Center, Hong Kong Eye Hospital, Hong Kong, China; 10 Department of Ophthalmology and Visual Sciences, The Chinese University of Hong Kong, Hong Kong, China; 11 Center for the Neural Basis of Cognition, University of Pittsburgh and Carnegie Mellon University, Pittsburgh, PA, USA; 12 Center for Neuroscience Imaging Research, Institute for Basic Science, Suwon, Korea; 13 Department of Biomedical Engineering, Sungkyunkwan University, Suwon, Korea; 14 Clinical and Translational Science Institute, University of Pittsburgh, Pittsburgh, PA, USA

## Abstract

The microstructural organization and composition of the corneoscleral shell (CSS) determine the biomechanical behavior of the eye, and are important in diseases such as glaucoma and myopia. However, limited techniques can assess these properties globally, non-invasively and quantitatively. In this study, we hypothesized that multi-modal magnetic resonance imaging (MRI) can reveal the effects of biomechanical or biochemical modulation on CSS. Upon intraocular pressure (IOP) elevation, CSS appeared hyperintense in both freshly prepared ovine eyes and living rat eyes using T2-weighted MRI. Quantitatively, transverse relaxation time (T2) of CSS increased non-linearly with IOP at 0–40 mmHg and remained longer than unloaded tissues after being unpressurized. IOP loading also increased fractional anisotropy of CSS in diffusion tensor MRI without apparent change in magnetization transfer MRI, suggestive of straightening of microstructural fibers without modification of macromolecular contents. Lastly, treatments with increasing glyceraldehyde (mimicking crosslinking conditions) and chondroitinase-ABC concentrations (mimicking glycosaminoglycan depletion) decreased diffusivities and increased magnetization transfer in cornea, whereas glyceraldehyde also increased magnetization transfer in sclera. In summary, we demonstrated the changing profiles of MRI contrast mechanisms resulting from biomechanical or biochemical modulation of the eye non-invasively. Multi-modal MRI may help evaluate the pathophysiological mechanisms in CSS and the efficacy of corneoscleral treatments.

The sclera and cornea are dense and fibrous connective tissues that form the outer coat of the eye, which act to support and protect the eye from the surrounding environments. These load-bearing connective tissues can dynamically interact with changing physiological conditions^1^,^2^. For example, varying intraocular pressures (IOP) alter the fiber orientations^3^,^4^ and collagen crimp structures^5^ in the corneoscleral shell and modify the biomechanical properties including strain, stiffness and hysteresis of the eye^6^. The corneoscleral shell in turn transfers tensile, compression and shear stresses on the lamina cribrosa in response to the IOP changes^7^, which contributes to the deformation of the optic nerve head and may result in neurodegeneration in the visual system in glaucoma^8^. The extracellular matrix in the corneoscleral shell may also be remodeled in other conditions such as aging^9^, myopia^10^ and keratoconus^11^, and plays an important role in transcorneal and transscleral drug delivery^12^,^13^. Having a non-invasive and non-destructive tool to better understand the microstructural organization and compositions of the corneoscleral shell in different environments can help determine the dynamics and the biomechanical and biochemical properties of the eye. Such knowledge is important for resolving the mechanisms of vision-related diseases involving the sclera and cornea, and may guide strategies for more effective drug delivery and corneoscleral treatment for vision preservation and restoration.

The corneoscleral shell structure has been studied extensively using atomic force^14^, transmission^15^ and scanning electron microscopies^16^, polarized light^5^, bright field^10^ and nonlinear microscopies^17^, as well as small-angle light scattering^18^ and small and wide-angle x-ray scattering^19^,^20^,^21^,^22^. Although these *ex vivo* imaging techniques have provided fundamental information on the microarchitecture and biochemistry of the tissue microstructures and collagen across ocular tissues with excellent resolution and sensitivity^7^, most of these techniques require chemical tissue processing or tissue sectioning, which is highly invasive and can be destructive or affect the true morphology of the collagen (e.g. substantial tissue shrinkage during preparation for histological labeling). These methods tend to have limited depth penetration, precluding analysis of the whole eye. Methods with wide fields of view, such as small-angle light scattering, have low resolution in the order of hundreds of microns, whereas methods with high resolution at sub-micron scale, such as transmission electron microscopy, have small fields of view, even when coupled with modern block-imaging techniques^23^,^24^. Altogether, these limitations prevent multi-modality assessments on the same sample, and prevent *in vivo* applications. Limited methods have been available for non-invasive and quantitative 3D assessments of the sclera and cornea in the whole globe^25^, and the exact contributions of the microstructural organization and composition of the corneoscleral shell to the pathogenesis of vision-related diseases remains largely undetermined.

Magnetic resonance imaging (MRI) offers non-invasive and multi-parametric methods for assessing the impaired visual system without depth limitation^26^,^27^,^28^,^29^,^30^,^31^,^32^,^33^,^34^. However, MRI studies of the corneoscleral shell have been limited, partly due to the intrinsically fast transverse magnetic resonance relaxation of the fibrous tissues and the resulting low MRI signal intensities available for biological examinations. Recently, our group demonstrated the use of the magic-angle enhancement effect to improve MRI sensitivity for detecting the structural details of the collagen-rich sclera and cornea tissues, and their changes in T2 and T2* transverse relaxation times in tissues fixed at various levels of IOP^25^. To date, it remains unclear how the MRI contrast mechanisms of fresh sclera and cornea tissues may respond dynamically with changing IOP, and whether the magic-angle effect can be applied to the living tissues to differentiate the biological conditions between normotensive and hypertensive eyes *in vivo*. Recent studies also suggested the possible role of scleral treatment as potential therapeutics to glaucoma^35^. However, its effectiveness on neuroprotection remains controversial, partly because of the lack of tools to characterize the biomechanical properties of the sclera *in vivo*, and to monitor their global and regional changes under stress or treatments.

Herein we report the effects of stepwise IOP changes on the fibrous tissues of freshly prepared, unfixed ovine eyes by examining the corresponding dynamic changes in T2 relaxation time and T2-weighted MRI signal intensities in the corneoscleral shell. In addition, we demonstrated *in vivo* the effects of chronic IOP elevation on the MRI contrasts in the sclera using a hydrogel-induced rat model of experimental glaucoma and T2-weighted MRI. Apart from basic MR relaxometry of the collagen-rich fibers^36^, advanced imaging techniques such as diffusion tensor MRI (DTI) and magnetization transfer MRI (MTI) can detect more specifically how collagen fibers are organized microstructurally, and how the extracellular matrix can be remodeled^37^,^38^. DTI measures water diffusion patterns in free, restricted or hindered compartments, and can reveal microstructural organization such as primary fiber orientation, directionality and directional diffusivities. MTI measures the magnetization transfer between free water and water bounded to macromolecules through chemical exchange and/or dipole-dipole interactions^39^, and can reflect changes in macromolecular structures and contents. The potentials of DTI and MTI for examinations of collagen-rich fibrous tissues have been demonstrated recently in studies of normal and diseased tendons^40^,^41^, menisci^42^, ligaments^43^,^44^ and cartilages^38^,^45^ but have not been investigated in the sclera or cornea for ophthalmic research. Such examinations are particularly important in diseases such as glaucoma, where vision loss cannot be recovered. In this study, we tested the central hypothesis that multi-modal MRI can detect and differentiate the effects of biomechanical or biochemical modulation on the sclera and cornea via IOP loading (mimicking ocular hypertension^46^), collagen cross-linking (mimicking aging conditions^47^ and corneoscleral stiffening^2^,^48^) and glycosaminoglycan cleavage (mimicking pathological changes in extracellular matrix compositions^49^,^50^,^51^). We used T2-weighted MRI, DTI and MTI to characterize the sclera and cornea with and without different levels of acute and chronic IOP loading. Furthermore, we tested the feasibility and sensitivity of T2-weighted MRI, DTI and MTI profiling to assess the treatment effects of cross-linking and glycosaminoglycan depletion on the sclera and cornea with different concentrations of glyceraldehyde and chondroitinase-ABC solutions, respectively.

## Results

### Effects of dynamic IOP changes on corneoscleral tissues by *ex vivo* T2 magnetic resonance relaxometry

**Figure 1:**
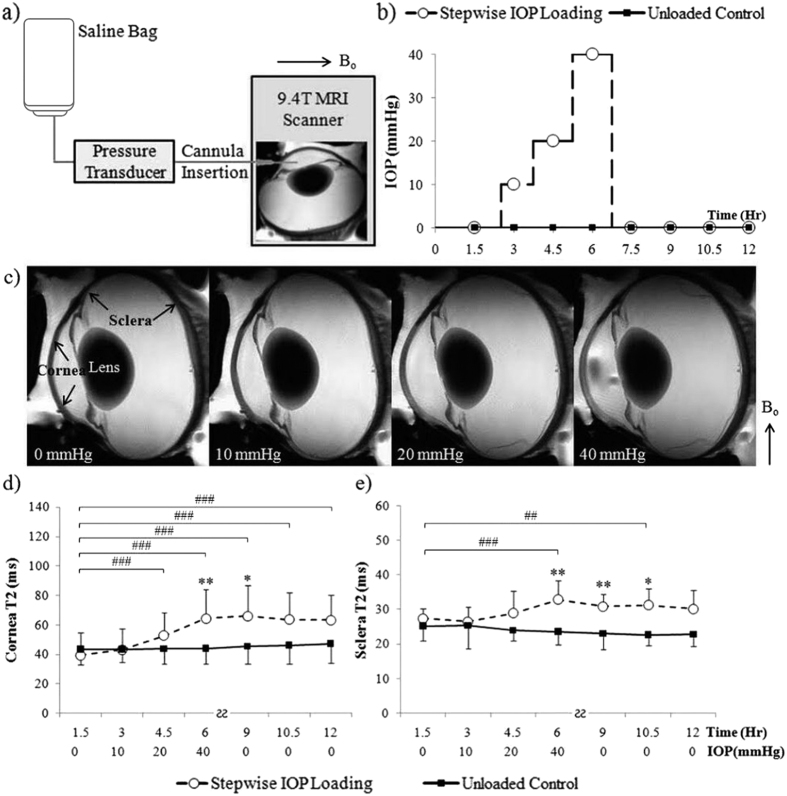
Dynamic imaging of the effects of stepwise intraocular pressure (IOP) changes on magnetic resonance relaxometry in the corneoscleral tissues of freshly prepared ovine eyes. (**a**) Schematic diagram of anterior chamber perfusion to a fresh, unfixed ovine eye in the 9.4 Tesla MRI scanner. A plastic cannula was inserted into the anterior chamber and connected to a saline bag that was risen at different heights to induce different levels of IOP elevation. (**b**) Experimental paradigm of the stepwise IOP loading MRI experiment and the IOP-unloaded control experiment. (**c**) Representative *ex vivo* T2-weighted MRI (T2WI) of the same ovine eye loaded at 0, 10, 20 and 40 mmHg. (**d,e**) Quantitative comparisons (mean ± standard deviation) of transverse relaxation time (T2) in cornea (**d**) and sclera (**e**) upon stepwise IOP loading (dashed lines) and in unloaded control (solid lines). Both cornea and sclera T2 gradually increased as IOP increased from 0 to 40 mmHg (Tukey’s multiple comparisons tests between first MRI session and subsequent sessions, ^##^p < 0.01, ^###^p < 0.001; comparisons between other sessions are not shown here for clarity) but remained unchanged after being unpressurized (Tukey’s multiple comparisons tests between MRI sessions, p > 0.05). No significant change was observed in the unloaded control tissues over time (Tukey’s tests between MRI sessions, p > 0.05) (Sidak’s multiple comparison tests between IOP loaded and unloaded control tissues: *p < 0.05, **p < 0.01) (B_o_: main magnetic field).

Figure 1 shows the effects of stepwise changes in IOP on the transverse relaxation time (T2) in the sclera and cornea of freshly prepared ovine eyes (Group 1). When IOP initially rose from 0 to 10 mmHg, no apparent T2 change was observed in either the sclera or the cornea. T2 began to increase when IOP was elevated from 10 to 20 mmHg. At 20 to 40 mmHg, T2 in sclera and cornea further increased and was significantly higher than the unloaded control. After being unpressurized from 40 mmHg back to 0 mmHg, T2 values of the ocular tissues remained higher than the unloaded control for about 6 hours.

### Effects of chronic IOP elevation on rat scleral tissues by *in vivo* T2-weighted MRI

**Figure 2:**
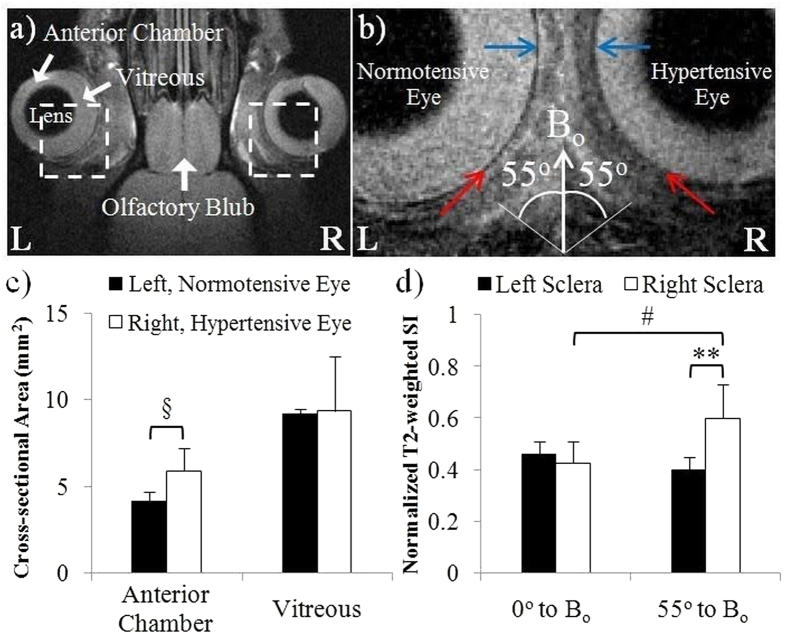
*In vivo* imaging of the effects of chronic IOP elevation on the rat scleral tissues. **(a**) Representative *in vivo* T2-weighted MRI (T2WI) of the hypertensive right eye and normotensive left eye of adult Long Evans rats at 1.5 weeks after intracameral hydrogel injection to the right eye. **(b)** Enlarged view of the rat eyes from the broken-line boxes in (**a**). Blue and red arrows in both eyes indicated the sclera tissues oriented at 0° to the main magnetic field (B_o_) and near the magic angle at 55° to B_o_ respectively. **(c**) Quantitative comparisons (mean ± standard deviation) of the cross-sectional areas of the anterior chamber and vitreous between the hydrogel-injected right eye and the uninjected left eye. **(d)** T2-weighted signal intensities (SI) in the sclera tissues oriented at about 0° or 55° (the magic angle) to B_o_ in both eyes. (Sidak’s multiple comparisons tests between left and right eyes: ^§^p < 0.05; **p < 0.01; Tukey’s multiple comparisons tests between 0° to B_o_ and 55° to B_o_ on the same eye: ^#^p < 0.05).

The effects of IOP loading on the ocular morphology and T2-weighted signal intensity in the living animals were illustrated in Fig. 2. In the rat model of experimental glaucoma, intracameral hydrogel injection to the right eye induced sustained IOP elevation from 2 days post-hydrogel injection up to MRI experiment at 1.5 weeks post-hydrogel injection. The IOP levels immediately before MRI experiment were 28.6 ± 4.7 mmHg in the right, hydrogel-injected eye and 13.7 ± 3.2 mmHg in the left, untreated control eye (Two-tailed paired t-test: p < 0.001). The right anterior chamber significantly enlarged at 1.5 weeks after hydrogel injection with no significant difference in the vitreous size between contralateral eyes (Fig. 2a,c). In addition, the sclera of the hypertensive right eye showed significantly higher normalized T2-weighted signal intensity than the normotensive left eye near the magic angle at 55° to the main magnetic field (B_o_) but not at 0° to B_o_ (Fig. 2b,d).

### Tissue magnetic resonance relaxation, diffusion and magnetization transfer properties in unloaded eyes by T2-weighted MRI, diffusion tensor MRI and magnetization transfer MRI

**Figure 3:**
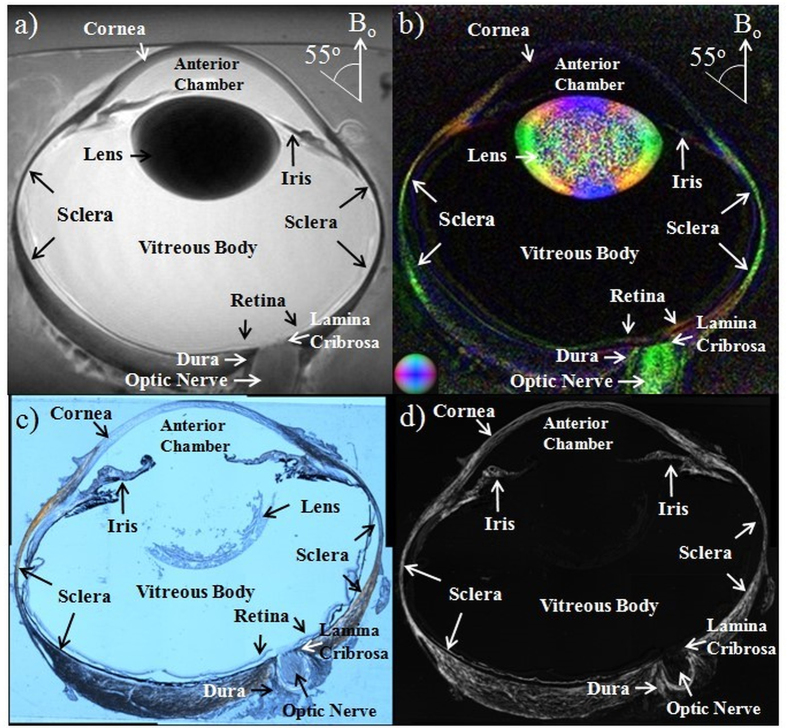
Whole eye imaging and histological confirmation. Representative **(a)** T2-weighted MRI, **(b)** color-encoded fractional anisotropy map in diffusion tensor MRI (DTI), **(c)** histological section imaged with polarized light microscopy, and **(d)** energy, or intensity-map of the collagen density parallel to the plane of the section, calculated from a series of polarized light microscopy images of the unloaded ovine eye. (Color representations for the principal diffusion directions in the color-encoded fractional anisotropy map: Blue: caudal-rostral; red: left-right; green: dorsal-ventral) (B_o_: main magnetic field).

In addition to T2 relaxometry, MRI allows multi-modal profiling among different structures in the whole globe of the eye. Within the unloaded ovine eye (Group 2), the lens and sclera appeared the darkest in T2-weighted MRI followed by the cornea, iris and optic nerve, whereas the anterior chamber and vitreous appeared the brightest (Fig. 3a). In the color-encoded fractional anisotropy map of the same eye in DTI (Fig. 3b), the principal diffusion orientations of the fibrous tissues including the sclera, cornea, lens cortex, retina, optic nerve and dura generally corroborated the fiber arrangements in the polarized light microscopy images of histological sections (Fig. 3c,d). In addition, a sharp boundary was observed in the color-encoded fractional anisotropy map between the outer cortex and inner nucleus of the lens, as well as near the lamina cribrosa in the optic nerve head. Quantitative measurements of the T2 relaxation times, diffusion properties and magnetization transfer properties of the fresh ovine eyes at 10 mmHg IOP were tabulated in Table 1 showing distinct MRI contrasts among cornea, sclera, retina, optic nerve, lens, anterior chamber and vitreous body. Specifically, the sclera had the lowest T2 value and the highest magnetization transfer ratio among all ocular components measured. The anterior chamber and vitreous body had the highest T2 and mean diffusivity, but the lowest magnetization transfer ratio and fractional anisotropy. The optic nerve and the lens cortex had the highest fractional anisotropy, while the optic nerve had the lowest mean diffusivity among all measured ocular components (Tukey’s multiple comparisons tests: p < 0.05).

### Microstructural organization and macromolecular contents of loaded and unloaded sclera, cornea and tendon tissues by T2-weighted MRI, diffusion tensor MRI and magnetization transfer MRI

**Figure 4:**
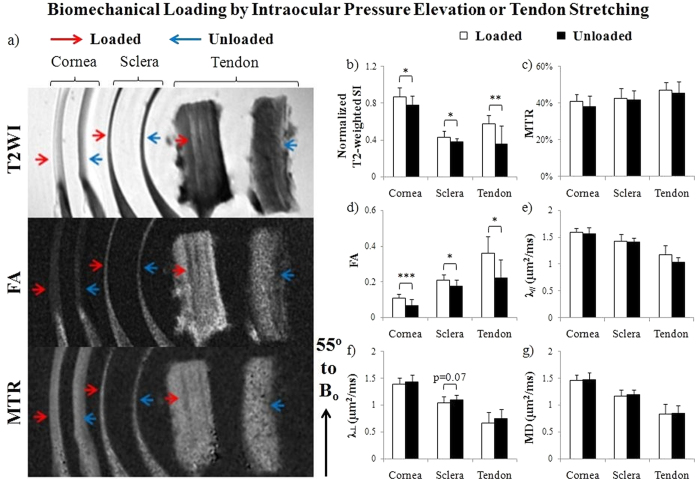
Microstructural organization and macromolecular contents of IOP-loaded sclera and cornea tissues, and stretch-loaded tendon tissues by T2-weighted MRI (T2WI), diffusion tensor MRI (DTI) and magnetization transfer MRI (MTI). (**a**) Representative T2WI (top), fractional anisotropy (FA) maps (middle) and magnetization transfer ratio (MTR) maps (bottom) of loaded (red arrows) and unloaded (blue arrows) cornea, sclera and tendon tissue strips. The tissues were oriented and measured near the magic angle at ~55° to the main magnetic field (B_o_) to enhance MRI signals for more sensitive examinations; (**b–g**) Quantitative comparisons (mean ± standard deviation) of (**b**) T2-weighted signal intensity (SI), (**c**) MTR, (**d**) FA, (**e**) axial diffusivity (λ_//_), (**f**) radial diffusivity (λ_⊥_) and (**g**) mean diffusivity (MD) between loaded and unloaded cornea, sclera and tendon strips. ANOVA tests showed significant differences between cornea, sclera and tendon tissues under the same loading conditions for each MRI parameter in (**b–g**) (p < 0.05) except loaded tissues in MTR (**c**) (p > 0.05) (Paired t-tests between loaded and unloaded cornea or sclera: *p < 0.05, **p < 0.01; ***p < 0.001; Unpaired t-tests between loaded and unloaded tendon: *p < 0.05).

In the tissue strips of the loaded and unloaded ovine eyes and tendons (Group 2) (Fig. 4), different profiles of T2-weighted signal intensity, fractional anisotropy and magnetization transfer ratio were observed in T2-weighted MRI, DTI and MTI among loaded and unloaded sclera, cornea and tendon when the tissues were oriented near the magic angle at about 55° to B_o_. In Fig. 4b, normalized T2-weighted signal intensities were higher in the cornea than the sclera and tendon under both loaded and unloaded conditions. In addition, all cornea, sclera and tendon tissues became hyperintense in T2-weighted MRI after loading similar to our previous study^25^. In Fig. 4c, the unloaded tendon exhibited the highest magnetization transfer ratio followed by the unloaded sclera and cornea. However, no significant difference in magnetization transfer ratio was found between loaded and unloaded tissues. DTI quantitation in Fig. 4d–g showed the highest fractional anisotropy and the lowest directional diffusivities (λ_//_ and λ_⊥_) in unloaded tendon followed by unloaded sclera and cornea. Loaded sclera, cornea and tendon tissues also showed significantly higher fractional anisotropy than unloaded tissues. A trend of lower λ_⊥_ was observed in loaded sclera than unloaded sclera.

### Effects of collagen crosslinking or glycosaminoglycan depletion on sclera and cornea tissues by T2-weighted MRI, diffusion tensor MRI and magnetization transfer MRI

**Figure 5:**
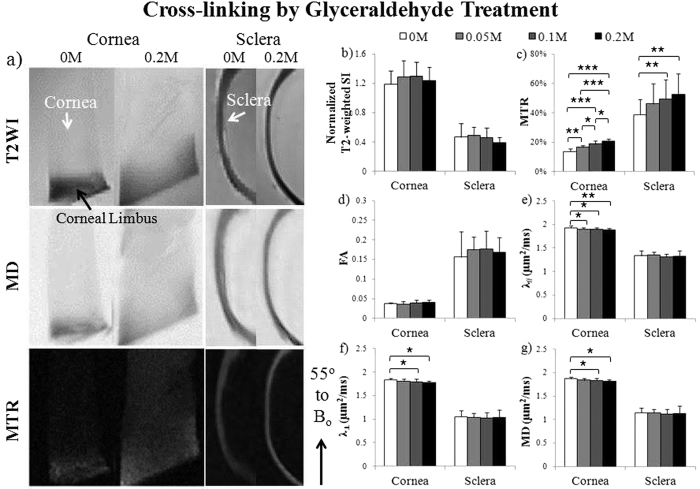
Microstructural and macromolecular changes in the fresh sclera and cornea tissues after glyceraldehyde cross-linking treatment with T2-weighted MRI (T2WI), diffusion tensor MRI (DTI) and magnetization transfer MRI (MTI). (**a**) Representative T2WI (top), mean diffusivity (MD) maps (middle) and magnetization transfer ratio (MTR) maps (bottom) of cross-linked cornea (left panel) and sclera (right panel) after treating with 0.2 M glyceraldehyde solution or sham solution (0 M). The tissue strips were oriented and measured near the magic angle at ~55° to main magnetic field (B_o_); (**b–g**) Quantitative comparisons (mean ± standard deviation) of (**b**) T2-weighted signal intensity (SI), (**c**) MTR, (**d**) fractional anisotropy (FA), (**e**) axial diffusivity (λ_//_), (**f**) radial diffusivity (λ_⊥_) and (**g**) MD in cornea and sclera strips after treatments with glyceraldehyde cross-linking solutions at 0, 0.05, 0.10 and 0.20 M concentrations. (Tukey’s multiple comparisons tests: *p < 0.05, **p < 0.01, ***p < 0.001).

**Figure 6:**
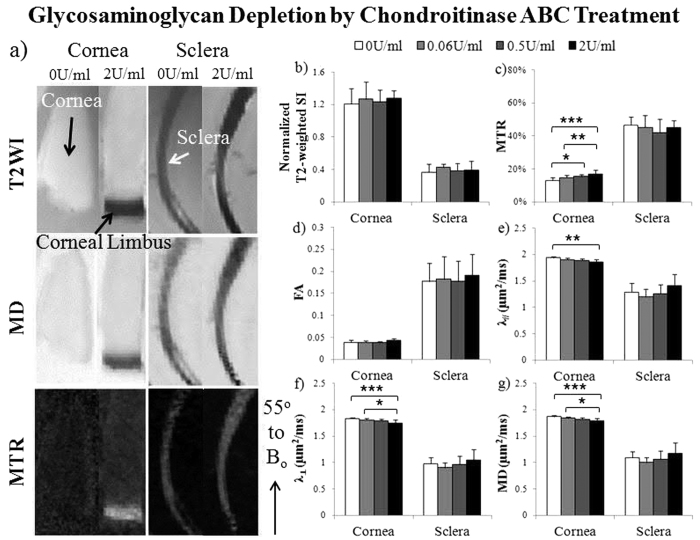
Microstructural and macromolecular changes in the fresh sclera and cornea tissues after glycosaminoglycan depletion by chondroitinase-ABC with T2-weighted MRI (T2WI), diffusion tensor MRI (DTI) and magnetization transfer MRI (MTI). (**a**) Representative T2WI (top), mean diffusivity (MD) maps (middle) and magnetization transfer ratio (MTR) maps (bottom) of glycosaminoglycan removal in cornea (left panel) and sclera (right panel) after treating with 2 units/ml (U/ml) of chondroitinase-ABC solution or sham solution (0 U/ml). Tissue strips were oriented and measured about the magic angle at ~55° to main magnetic field (B_o_); (**b–g**) Quantitative comparisons (mean ± standard deviation) of (**b**) T2-weighted signal intensity (SI), (**c**) MTR, (**d**) fractional anisotropy (FA), (**e**) axial diffusivity (λ_//_), (**f**) radial diffusivity (λ_⊥_) and (**g**) mean diffusivity (MD) in cornea and sclera strips after treatment with chondroitinase-ABC solutions at concentrations of 0, 0.06, 0.5 and 2 U/ml. (Tukey’s multiple comparisons tests: *p < 0.05, **p < 0.01, ***p < 0.001).

When the fresh ovine cornea and sclera tissues were treated with increasing concentrations of glyceraldehyde cross-linking solutions (Group 3a), the occurrence of non-enzymatic glycation was clearly evident by a gradual color change from whitish to brownish yellow similar to a recent study^52^. In MTI, magnetization transfer ratio in both cornea and sclera tissues significantly increased with increasing glyceraldehyde concentrations (Fig. 5c), whereas DTI quantitation in Fig. 5d–g showed that λ_//_, λ_⊥_ and mean diffusivity in cornea significantly decreased with increasing glyceraldehyde concentrations. Similar changes were detected in chondroitinase-ABC treated ovine cornea but not sclera (Group 3b), whereby significantly increasing magnetization transfer ratio and decreasing λ_//_, λ_⊥_ and mean diffusivity were observed with increasing chondroitinase-ABC concentrations (Fig. 6). No significant difference in fractional anisotropy or normalized T2-weighted signal intensity was detected in the cornea or sclera among different glyceraldehyde or chondroitinase-ABC concentrations.

## Discussion

In this study, we used freshly prepared ovine eyes to evaluate the tissue magnetic resonance relaxation in response to dynamic IOP changes, since tissue fixation may alter the MRI contrast mechanisms in the fibrous tissues^37^ as well as the biomechanical responses of the eye to changing IOP^1^,^2^. When the fresh ocular tissues were loaded beyond physiological IOP levels, the consistent T2 increase observed in Fig. 1 might be explained by the diminishing magnetization spin dephasing between increasingly distant neighboring protons during collagen fiber straightening^36^. More importantly, the non-linearity of T2 changes with increasing IOP at 0–40 mmHg echoed with recent histological studies showing non-linear uncrimping of collagen fibrils in the corneoscleral shell with IOP loading^53^,^54^. After being unpressurized, T2 of the fresh IOP-loaded ovine ocular tissues remained significantly higher than the unloaded control for few hours, suggestive of hysteresis or hydraulic effect after IOP loading^55^. MRI detection of hysteresis is potentially important to determine globally the biomechanical state of the corneoscleral shell and its interplay with the surrounding ocular structures and the behavioral outcomes.

Apart from dynamic MRI assessments of fresh ocular tissues *ex vivo*, the present study demonstrated the use of *in vivo* magic angle-enhanced MRI to differentiate sclera tissues between normotensive and hypertensive eyes in the rat glaucoma model of chronic IOP elevation as shown in Fig. 2. These initial results opened up the possibility for future non-invasive MRI assessments of the corneoscleral biomechanics in the living eyes, which may help better predict the dynamic profiles of IOP and identify new modifiable risk factors for glaucomatous optic neuropathy.

In addition to the sclera and cornea, multi-modal MRI allows non-invasive profiling and comparisons among different structures in the whole globe of the eye without depth limitation. In the normal ovine eye in Fig. 3 and Table 1, the short T2 and the resulting low T2-weighted signals in the lens and sclera could be explained by the abundance of tightly bound protons associated to macromolecules such as collagen. Furthermore, the decreasing T2-weighted signal intensities within the lens indicated increasing bound water and less free water from increasing protein concentration toward the center of the ocular lens^56^. The long T2 and bright T2-weighted signals in the anterior chamber and vitreous body reflected the abundance of mobile water protons in the aqueous humor and vitreous humor. Apart from conventional T2-weighted MRI, advanced diffusion MRI techniques such as DTI allow microstructural assessments by measuring water diffusion patterns in biological tissues. The diffusivity patterns observed in the ovine lens generally agreed with other species^56^, which showed anisotropic water movements and stronger diffusivity in the outer cortex containing elongated lens fiber cells, and isotropic patterns and weaker diffusivity in the protein core of the lens in the absence of a blood supply. The high fractional anisotropy in the intraorbital optic nerve indicated the unilaterality of myelinated axonal fibers, whereas the sharp boundary at the lamina cribrosa and between the outer cortex and inner nucleus of the lens in the fractional anisotropy map indicated the presence of a diffusion barrier limiting the transport of water and other metabolites across the boundary. While DTI may help examine ocular tissue microstructures, MTI may probe macromolecular contents in the ocular tissues. Although the short T2 of the less mobile protons associated with macromolecules may not be easily detected directly in conventional MRI, the coupling between the macromolecular protons and the mobile protons allows the spin state of the macromolecular protons to influence the spin state of the liquid protons through exchange processes^39^, leading to the magnetization transfer contrast. The magnetization transfer effect is dependent on the concentration, mobility, and surface chemistry of the macromolecules^57^. In the present study, the sclera, cornea, optic nerve and lens cortex had 6–10 times higher magnetization transfer ratio than the anterior chamber and vitreous body, indicative of the higher macromolecular contents in these ocular tissues compared to the water-rich ocular chambers. *In vivo* measurement of magnetization transfer ratio has been demonstrated to reflect the collagen status and may be applied for the routine evaluation of normal and abnormal fibrous tissues such as articular cartilage^58^. Previous MTI studies have shown that magnetization transfer can give better image contrasts between the lens cortex and nucleus than conventional T2-weighted MRI sequences in normal and cataratous lenses^57^. Taken together, combined T2-weighted MRI, DTI and MTI may offer promises to improve the specificity in evaluating the conditions of normal and abnormal eye globes.

To validate the feasibility and sensitivity of multi-modal MRI use in IOP-loaded and unloaded eyes, we compared the T2-weighted MRI, DTI and MTI profiles between IOP-loaded and unloaded cornea and sclera tissues in Fig. 4. Stretch-loaded and unloaded tendons were also scanned as a positive control given the similar structural compositions of the tendon and the corneoscleral shell as well as existing reports detailing MRI evaluations of the tendon^25^,^36^,^41^. In DTI, among the 3 unloaded tissues, the highest fractional anisotropy observed in tendon conformed with the nearly parallel alignment of tendon collagen fibrils to the long axis^59^. Although fibers in the corneoscleral shell are also highly aligned in-plane, they may traverse each other in slightly different directions to provide maximal mechanical strength within the curved globe^22^,^60^. Such crossing fibers may lead to lower overall fractional anisotropy in ocular tissues compared to the tendon. The highest directional diffusivities (λ_//_ and λ_⊥_) in the cornea followed by the sclera and tendon likely reflect the rich water content and more regular lattice arrangement in the corneal stroma. Upon pressure loading, the collagen-rich tissues experienced stretch and compression leading to fiber straightening^3^ and a more anisotropic microstructural environment^61^. Our results of the significantly higher fractional anisotropy but not directional diffusivities in loaded than unloaded tisues suggested that fractional anisotropy is a more sensitive DTI marker than λ_//_ and λ_⊥_ to detect pressure loading in ocular tissues and tendons. Previous studies suggested that the dynamic responses of the sclera to experimental glaucoma may be as important as the baseline anatomic features in explaining susceptibility to neuronal damage^61^. Here, the increase in fractional anisotropy of loaded sclera and cornea tissues may indicate short-term plasticity in the interwoven collagen lamellae in response to elevated IOP during glaucoma exposure^62^. In MTI, the magnetization transfer effect in the unloaded tendon was slightly stronger than the unloaded sclera and significantly stronger than the unloaded cornea. This may be explained by the most abundant collagen contents in tendon (~100% dry mass) compared to sclera and cornea (~70–90% dry weight)^63^. The insignificant difference in magnetization transfer ratio between loaded and unloaded tissues suggests no apparent alteration in macromolecular contents despite microstructural changes upon acute pressure loading^4^. Although ocular and tendon tissues have similar extracellular matrix compositions, there are some important differences in their structural hierarchies. Tendon has a highly ordered fascicular structural hierarchy whereas corneal and scleral collagen does not. The molecular tilt in the collagen fibrils are also different between the tissues, with a more oblique molecular tilt in the cornea (~15°) vs. the tendon (~4°) with respect to the fibril axis^19^. It has been suggested that this molecular tilt is a result of the lower axial periodicity of microfibrils in the cornea compared to the tendon^19^. The consequences of this difference in molecular tilt on our MRI results are unclear. In a previous MRI study examining the extents of T2 and T2* signal enhancement at multiple angles to the main magnetic field, we did not detect a deviation of angle at maximum signal enhancement from the magic angle between tissues^25^. Whether this tilt may have contributed to the lower magic-angle effect strength and the sensitivity to DTI and MTI changes in the ocular tissues compared with the tendon will be the subject of further investigation. It is critical to determine whether the corneoscleral reorganization in human eyes is a beneficial adaptation or a detrimental contributor to optic nerve head injury in glaucoma. Multi-modal MRI may be useful to measure the biomechanical behavior of the eyes non-invasively in future, both to monitor the baseline state of the eye as a risk factor for future development of glaucoma, and to assess disease progression.

MRI of ocular tissue structures and compositions may provide a non-destructive and non-invasive means for screening patients’ susceptibility to glaucomatous damage and for determining whether the greater stiffness of human glaucoma eyes is present at baseline, whether it develops as a response to the disease, or both^2^, in order to design optimal biomechanical modification therapies^35^. In MTI, it has been suggested that magnetization transfer ratio may differentiate between various pathomimetic degradative procedures^64^. While the baseline magnetization transfer ratio in fibrous tissues may be primarily due to the tissue collagen concentration, changes in magnetization transfer ratio may be due to physiological or pathophysiological changes in tissue structure and glycosaminoglycan concentration^58^. In the present study, the increased magnetization transfer ratio in the cross-linked cornea and sclera in Fig. 5 could be explained by the larger pool of immobile water molecules available for transfer of magnetization to free water when cross-linking packing density increased at a constant macromolecular concentration^37^. Glyceraldehyde cross-linking may also reduce permeability or induce tissue shrinkage leading to the reduced diffusivity observed in the cornea^65^. These findings introduced the possibility of multi-modal MRI to characterize the state of collagen cross-linking in different regions of the eye in future *in vivo* studies. Pathological changes in glycosaminoglycan content have been observed in eyes with glaucoma and myopia^49^,^50^,^51^, whereas glycosaminoglycan removal may contribute to an increase in hydration and altered creep and stiffness of the sclera^66^. In the present study, glycosaminoglycan depletion by chondroitinase ABC did not alter T2-weighted signals in cornea and sclera in Fig. 6 similar to a recent study on chondroitinase ABC-treated cartilage cultures with altered biomechanical properties but not collagen contents or T2 values^67^. On the other hand, chondroitinase ABC increased magnetization transfer ratio and decreased diffusivities but not fractional anisotropy in the cornea, whereas no significant change was observed in the sclera under the same chondroitinase-ABC concentrations. Whether the observed differences in treatment responses between cornea and sclera tissues were due to different extents of enzyme penetration, different tissue compositions or other factors will require additional studies in future employing a larger range of chondroitinase-ABC concentrations and considering other MRI modalities such as T1ρ and chemical exchange saturation transfer to further improve specificity. Complementary to other imaging modalities such as spectral-domain optical coherence tomography and ultrasound^7^, multi-modal MRI may further improve the understanding of the remodeling of the collagen and proteoglycan structures in the corneoscleral shell, and the biomechanical characteristics that predispose clinically to the development of ocular pathologies.

This study has several limitations. While this study demonstrated the feasibility and sensitivity to detect changes in MRI contrast mechanisms in response to biomechanical or biochemical manipulation in the eye, the direct linkages between MRI contrast mechanisms and biomechanical properties, despite demonstrated in other fibrous tissues under various conditions^68^,^69^, remain to be elucidated in the eye in the future. On the other hand, although our MRI methods had achieved sub-millimeter in-plane resolution to examine the collagen organization relevant to lamellar and crimp properties, the current MRI study is limited in its out-of-plane resolution due to the relatively large 1 mm slice thickness. It should be noted that this slice thickness was chosen as an early step to guarantee sufficient signal-to-noise ratio for detecting tissue changes in response to biomechanical or biochemical modulation. Since MRI has no depth limitation and the slice thickness can be further optimized, we expect that multi-modal MRI has the potential to resolve collagen organization in thinner slices for more detailed 3D analyses of the collagen structure. This may provide a more complete evaluation of the eye’s anisotropic structure and biomechanical performance in future studies. Lastly, while ovine eyes were chosen in this study for *ex vivo* exmainations to mimic human eyes of similar sizes, when translating this study to imaging humans or larger animals *in vivo*, a larger scanner bore size and lower magnetic field strengths are expected for more practical use, and limited image signal-to-noise ratio may be resulted at high resolutions. Eye motion is another potential challenge for *in vivo* ocular imaging. Using specialized orbital coils, recent studies had achieved sub-millimeter resolutions for human ocular MR imaging in the clinical 3-Tesla scanners and research-based 7-Tesla human scanners^70^,^71^. Eye tracking and gaze fixation can also help minimize motion artifacts and improve image quality within clinically acceptable scan time. By instructing our subject to maintain stable eye fixation on several targets at various angles relative to the main magnetic field, our pilot study demonstrated the magic-angle effect in the human sclera *in vivo* using 3-Tesla MRI^72^. These studies provide initial evidence to support the translation of the current high-field sclera and cornea MRI toward *in vivo* imaging in humans.

## Conclusion

T2-weighted MRI, DTI and MTI non-destructively distinguished between different ocular tissues with and without biomechanical or biochemical modulation via acute and chronic IOP loading, collagen crosslinking and glycosaminoglycan depletion. Multi-modal MRI may provide cross-sectional and longitudinal monitoring of the ocular fiber organization and remodeling in aging and diseases involving the corneoscleral shell. This may help evaluate the pathophysiological mechanisms in the corneoscleral shell and the efficacy of corneoscleral treatments in a variety of ophthalmic diseases.

## Materials and Methods

### Ovine eye and tendon tissue preparation for *ex vivo* imaging

Twenty three pairs of ovine eyes were obtained from the local abattoir and processed within 12 hours of death to minimize mechanical deterioration^73^. The ovine eyes were divided into 3 groups:

#### Group 1: Effects of dynamic biomechanical loading on fresh ovine eyes by T2 magnetic resonance relaxometry

Twelve fresh, unfixed ovine eyes underwent anterior chamber perfusion in the 9.4 Tesla MRI scanner using a plastic cannula connected to a saline bag (0.9% sodium chloride; Baxter International Inc., Deerfield, IL, USA) and a pressure transducer (BIOPAC Systems, Goleta, CA, USA) so as to image the dynamic effects of stepwise changes in IOP on the tissue transverse relaxation times (T2) of the corneoscleral shell in the same eyes (Fig. 1a,b). Six of these 12 ovine eyes were loaded at 0, 10, 20 and 40 mmHg by raising the saline bag at different heights consecutively inside the MRI scanner, followed by unpressurization back to 0 mmHg until the end of the experiment. Although 0 mmHg is not a normal physiological condition, it was selected as the lowest pressure to provide fundamental insights into the state of the tissues without the load from IOP, which is essential in the formulation of a comprehensive mechanistic model of the tissues^5^,^53^,^54^,^74^. The pressure transducer was used to ensure a constant pressure was applied at the desired level during each experimental session. Six other fresh ovine eyes were cannulated but kept at 0 mmHg throughout the entire MRI experiment as an unloaded control. Hydrogel (McNeil-PPC, Inc., Skillman, NJ, USA) was applied to keep the surface of the ovine eyes moist throughout the experiment.

#### Group 2: Effects of biomechanical loading on ovine cornea, sclera and tendon tissues by T2-weighted MRI, diffusion tensor MRI and magnetization transfer MRI

Eight ovine eyes were loaded at an IOP of 50 mmHg using a gravity perfusion fixation system with a cannula inserted into the anterior chamber^25^ outside the MRI scanner, so as to image the effects of IOP loading on the corneoscleral shell with T2-weighted MRI, DTI and MTI. Twelve additional ovine eyes were cannulated but unpressurized. Ten minutes later, all eyes were immersion fixed with 10% formalin for 24 hours while remained loaded or unloaded, and then washed in phosphate buffered saline (PBS) (Fisher Scientific Inc., Pittsburgh, PA, USA). Three of the 12 unloaded ovine eyes underwent whole-globe MRI while another unloaded eye was processed in sucrose, cryosectioned axially into 30 μm thick histological sections and imaged with polarized light microcopy^75^. The remaining 8 loaded and 8 unloaded eyes were dissected using razors and surgical scissors to isolate the sclera and cornea of 8–12 mm long, 2–5 mm wide and 2–3 mm thick. Given the similar structural compositions between the tendon and corneoscleral shell, 13 ovine Achilles tendons from the same set of animals were washed in PBS and dissected to isolate thin strips of similar dimensions to compare with the ocular tissues. Four tendon strips were randomly selected and loaded using alligator clips attached to a rod to keep stretched for 10 minutes^25^, followed by immersion fixation with 10% formalin for 24 hours while remaining stretched. The remaining 9 strips were fixed but unloaded.

#### Group 3: Effects of cross-linking and glycosaminoglycan depletion on ovine cornea and sclera tissues by T2-weighted MRI, diffusion tensor MRI and magnetization transfer MRI

Sixteen freshly prepared ovine eyes were divided into 2 sub-groups of 8 eyes each in order to image the effects of glyceraldehyde cross-linking (Group 3a) and glycosaminoglycan depletion by chondroitinase-ABC treatment (Group 3b) on the sclera and cornea with T2-weighted MRI, DTI and MTI. Each eye was dissected at the central globes using razors and surgical scissors to give 4 sclera and 4 cornea fresh tissue strips of similar dimensions as in Group 2. In Group 3a, the 4 sclera and 4 cornea strips from each eye were treated with D-(+)-glyceraldehyde (Sigma-Aldrich, St. Louis, MO, USA) solutions at 0, 0.05, 0.1, or 0.2 M at 37 °C in the incubator for 12 hr. In Group 3b, the 4 sclera and 4 cornea strips from each eye were treated with chondroitinase-ABC (Sigma-Aldrich, St. Louis, MO, USA) solutions at 0, 0.06, 0.5 or 2 unit/ml at 37 °C in the incubator for 12 hr. The different glyceraldehyde concentrations were prepared by diluting the solutions with 1x PBS at pH 7.4 (Fisher Scientific Inc., Pittsburgh, PA, USA), whereas the different chondroitinase-ABC concentrations were prepared by diluting the solutions with 1 M Tris-HCl at pH 8.0 (Mediatech, Inc, Manassas, VA, USA ), 3 M sodium acetate buffer solution (Sigma-Aldrich, St. Louis, MO, USA), and 0.1% bovine serum albumin solution (Fisher Scientific Inc., Pittsburgh, PA, USA). Samples treated with sham solution without glyceraldehyde or chondroitinase-ABC were indicated as 0 M or 0 unit/ml.

### Rat model preparation for *in vivo* imaging

All *in vivo* animal experiments were performed in accordance with the ARVO Statement for the Use of Animals in Ophthalmic and Vision Research and protocols reviewed and approved by the University of Pittsburgh’s Institutional Animal Care and Use Committee. Five adult female Long Evans rats were first anesthetized by inhaling a mixture of air and isoflurane (3% for induction and 1.25% for maintenance). Proparacaine (Bausch & Lomb, Inc., Rochester, NY, USA) was topically administered to anesthetize the surface of the eye. The right eye was then intracamerally injected with 20 μL of a solution containing 6% vinysulfonated hyaluronic acid and 6% thiolated hyaluronic acid by a microinjection system through a sharp glass micropipette (World Precision Instruments, Sarasota, FL, USA) under a surgical microscope. The hyaluronic acid derivatives were synthesized as reported previously^76^. The polymer mixture solidified shortly after injection to an optically clear cross-linked hydrogel causing aqueous outflow obstruction and sustained IOP elevation^77^. The left eye did not receive any injection and served as an internal control. The IOPs of both eyes were measured using the TonoLab rebound tonometer (Colonial Medical Supply, Franconia, NH, USA) under light isoflurane gas anesthesia. At least 18 valid, non-failed IOP values from each eye were obtained and averaged. IOP measurements were performed every 3–4 days after hydrogel injection.

### MRI protocol

All MRI experiments were performed using a 9.4-Tesla/31-cm Varian/Agilent horizontal MRI scanner (Santa Clara, CA, USA) with a 38 mm-diameter transmit-receive volume coil for *ex vivo* ovine eye and tendon imaging, and a volume transmit and surface receive coil for *in vivo* rat eye imaging.

For T2 relaxometry in Group 1, the whole globe of the cannulated fresh eye underwent T2 mapping at each pressure level using a spin-echo MRI pulse sequence with the following imaging parameters: Repetition time (TR)/echo time (TE) = 1000/9.48 ms, echo space time = 9.48 ms, number of echoes = 5, in-plane resolution = 130 × 130 μm^2^, slice thickness = 1 mm and number of repetitions = 2. Diffusion tensor MRI (DTI) and magnetization transfer imaging (MTI) were also performed at 10 mmHg using fast spin-echo sequences with the following imaging parameters: (i) DTI: 2 non-diffusion-weighted (b_0_) images and 12 diffusion weighted images with 12 gradient directions at diffusion weighting factor (b) = 1000 s/mm^2^, diffusion gradient duration time (δ)/diffusion gradient separation time (Δ) = 5/17 ms, TR/TE = 2300/27.8 ms, echo train length = 8 and number of repetitions = 1; (ii) MTI: 9.5 μT saturation pulses at +6000 Hz off-resonance and 150 ms pulse length, TR/TE = 1500/8.43 ms, echo train length = 8 and number of repetitions = 2. The MTI parameters were selected so as to minimize direct saturation of the bulk water^64^. Both DTI and MTI shared the same slice geometry, with in-plane resolution = 140 × 140 μm^2^ and slice thickness = 1 mm. The total scanning duration for each pressure level was limited to 1 hr, and MRI scans began at about 20 min after one stepwise change in pressure to allow the ocular tissues to reach viscoelastic equilibrium^78^.

In Group 2, DTI was performed to the whole globe of the fixed, unloaded eye under similar DTI parameters as in Group 1 except b = 500 s/mm^2^, δ/Δ = 5/12 ms, TR/TE = 2300/21.6 ms and number of repetitions = 4. The sectioned eye and tendon tissue samples in Groups 2 and 3 were suspended in agarose gel and were orientated near the magic angle at ~55° to the main magnetic field (B_o_) during imaging. DTI and MTI were performed to the sectioned tissues with the same imaging parameters as in Group 1 except number of repetitions = 4 for DTI and in-plane resolution = 125 × 125 μm^2^ for both DTI and MTI.

For the rat model of experimental glaucoma, T2-weighted MRI was acquired at 1.5 weeks after induction of chronic IOP elevation under ketamine (75 mg/kg) and xylazine (10 mg/kg) anaesthesia using a spin-echo imaging sequence with TR/TE = 1000/13.6 ms, in-plane resolution = 55 × 55 μm^2^, slice thickness = 1.0 mm, number of repetitions = 2 and total scanning duration = 17 min.

### Data analysis

In Group 1, the T2 transverse relaxation times of the eyes at each pressure level were derived from curve fitting of the T2-weighted signal decay with echo time using the equation S(TE) = S(0)e^(−TE/T2)^ where S is the signal intensity (S) from the spin-echo MR image at a particular echo time (TE)^25^. T2-weighted MRI in Group 1 was obtained from T2 mapping, whereas T2-weighted images in Groups 2 and 3 were obtained from the non-diffusion-weighted b_0_ images in DTI. For DTI, co-registration between non-diffusion-weighted b_0_ images and diffusion-weighted images was performed using SPM8 (Wellcome Department of Imaging Neuroscience, University College, London, UK). Using DTIStudio v3.02 (Johns Hopkins University, Baltimore, MD), 3 × 3 diffusion tensors were fitted on a pixel-by-pixel basis from the non-diffusion-weighted b_0_ images and the diffusion-weighted images. The eigenvectors and eigenvalues of the diffusion tensors were derived to compute the DTI parametric maps including the fractional anisotropy directionality color map, fractional anisotropy value map, and axial diffusivity (λ_//_), radial diffusivity (λ_⊥_) and mean diffusivity maps. For MTI, magnetization transfer ratio was calculated by the equation (M_0_ − M_sat_)/M_0_ where M_sat_ and M_0_ are the magnetization signals with and without saturation pulse respectively. Regions of interest were manually drawn and measured on the cornea, sclera or tendon near the magic angle at ~55° to the main magnetic field (B_o_), as well as the retina, optic nerve, lens cortex, anterior chamber, vitreous body using ImageJ v1.47 (Wayne Rasband, NIH, USA) for quantitative analyses. T2-weighted signal intensities in the eye or tendon tissues were normalized to the nearby gel for the ovine in Groups 2 and 3, and to the nearby vitreous for rats to account for coil sensitivity and magnetic field inhomogeneity. Results were presented as mean ± standard deviation. Comparisons among conditions were performed using analyses of variance (ANOVA) followed by post-hoc multiple comparisons tests using GraphPad Prism v6.00 (GraphPad Software Inc., La Jolla, CA, USA) unless otherwise specified. Results were considered significant when p < 0.05.

## Additional Information

**How to cite this article**: Ho, L. C. *et al*. Non-invasive MRI Assessments of Tissue Microstructures and Macromolecules in the Eye upon Biomechanical or Biochemical Modulation. *Sci. Rep*. **6**, 32080; doi: 10.1038/srep32080 (2016).

## Figures and Tables

**Table 1:** Summary of T2-weighted MRI, magnetization transfer MRI (MTI) and diffusion tensor MRI (DTI) measurements in different components of the fresh ovine eyes at the physiological intraocular pressure of 10 mmHg (mean ± standard deviation).

	T2 (ms)	MTR	FA	λ_//_ (μm^2^/ms)	λ_⊥_ (μm^2^/ms)	MD (μm^2^/ms)
Cornea	43.3 ± 14.1	26.6% ± 4.8%	0.126 ± 0.038	1.638 ± 0.068	1.375 ± 0.121	1.463 ± 0.102
Sclera	26.5 ± 4.2	43.1% ± 8.4%	0.386 ± 0.061	1.270 ± 0.067	0.738 ± 0.078	0.915 ± 0.064
Retina	122.5 ± 18.3	15.1% ± 4.8%	0.283 ± 0.152	0.565 ± 0.053	0.380 ± 0.096	0.441 ± 0.068
Optic Nerve	51.3 ± 7.9	40.5% ± 5.3%	0.513 ± 0.105	0.452 ± 0.116	0.195 ± 0.024	0.281 ± 0.047
Lens Cortex	33.9 ± 7.7	40.0% ± 7.4%	0.597 ± 0.050	1.289 ± 0.097	0.464 ± 0.069	0.739 ± 0.075
Anterior Chamber	188.6 ± 36.9	4.0% ± 0.7%	0.063 ± 0.003	2.144 ± 0.081	1.956 ± 0.068	2.019 ± 0.072
Vitreous Body	189.4 ± 12.8	3.8% ± 0.5%	0.065 ± 0.003	2.140 ± 0.025	1.945 ± 0.017	2.010 ± 0.019

(T2: transverse relaxation time; MTR: magnetization transfer ratio; FA: fractional anisotropy; λ_//_: axial diffusivity; λ_⊥_: radial diffusivity; MD: mean diffusivity).
